# Methodological flaws on “manual therapy for the pediatric population: a systematic review” by Prevost et al. (2019)

**DOI:** 10.1186/s12906-020-03145-6

**Published:** 2021-01-05

**Authors:** Hainan Yu, Heather Shearer, Anne Taylor-Vaisey, Silvano Mior, Leslie Verville, Gaelan Connell, Pierre Côté

**Affiliations:** 1 Centre for Disability Prevention and Rehabilitation at Ontario Tech University and Canadian Memorial Chiropractic College, 6100 Leslie Street, Toronto, Ontario M2H 3J1 Canada; 2 Faculty of Health Sciences, Ontario Tech University, 2000 Simcoe Street North, Oshawa, Ontario L1H 7L7 Canada; 3 Canadian Memorial Chiropractic College, 6100 Leslie Street, Toronto, Ontario M2H 3J1 Canada; 4 Institute of Health Policy, Management and Evaluation, University of Toronto, 155 College Street, Toronto, Ontario M5T 3M7 Canada; 5 Canada Research Chair in Disability Prevention and Rehabilitation, Ontario Tech University, 2000 Simcoe Street North, Oshawa, Ontario L1H 7L7 Canada

**Keywords:** Pediatric, Manual therapy, Manipulation, Mobilization, Systematic review

## Abstract

Prevost et al. published a systematic review evaluating the use of manual therapy for clinical conditions in the pediatric population in 2019. However, several methodological flaws in the conduct of the review limit the internal validity of its conclusions. We caution readers about the validity of the recommendations and suggest that the review not be used to inform the clinical management of pediatric patients.

## Background

We reviewed the systematic review by Prevost et al [1] and are concerned about its methodological quality. We critically appraised the systematic review using the Scottish Intercollegiate Guidelines Network (SIGN) criteria for systematic reviews. Our appraisal of the review suggests that it suffers from numerous methodological flaws, and that it does not meet the minimal quality criteria of a valid systematic review. The methodological limitations refer to the formulation of its research question, the search strategy, the classification of the quality of evidence, and the evidence synthesis. These limitations restrict the interpretation and generalizability of this review.

## Methodological flaws

Specifically, our first concern is with the stated objectives of the review: “*…to evaluate the use of manual therapy for clinical conditions in the pediatric population, assess the methodological quality of the studies found, and synthesize findings based on health condition.”* These objectives are vague and do not comply with accepted frameworks to formulate research questions to assess the effectiveness of interventions [2, 3] In particular, the clinical conditions should be identified in the research question since other conditions (e.g., cardiovascular disease, renal disease) were not included in the review. “Assess the methodological quality of the studies found” is not a research question/objective. Furthermore, “Synthesize findings based on health condition” is not clear, specific or representative, or reproducible.

Second, the search strategy presented in Table 1 is not comprehensive and does not meet the current standards for systematic review literature searching, as defined in the Peer Review of Electronic Search Strategies (PRESS) [4] Specifically, important search terms that could identify studies conducted in the pediatric populations were not included (e.g., newborn, infant, youth). Moreover, according to PRESS, the search strategy should be developed with the assistance of a health sciences librarian and reviewed by a second health sciences librarian using the PRESS checklist [5] Although the authors mention that three librarians were used, it is unclear whether and how the librarians reviewed each other’s work using the PRESS checklist. Furthermore, the authors claimed that they searched several databases (as listed in Table 1). However, we are concerned about the validity of this statement because ScienceDirect and McCoy Press are journal publishers, not databases. Therefore, we cannot exclude that the search strategy led to significant selection bias in the included literature.

Third, this review used cut-off points to classify the quality of evidence (i.e., “low quality study if the score was between 0 and 33.3%, medium quality if the score was between 33.4 and 66.6%, and high quality if the score was above 66.6%”). Although cut-points are commonly used to classify levels of evidence, it is important to understand the limitations of this methodology. One important limitation is that impact of biases identified by the items is not weighted (i.e., each item is given the same weight regardless of their impact on study results) [6] Therefore, a study with a high score can be deemed to be of high quality even if it suffers from a fatal methodological flaw (e.g., differential attrition rates leading to differential ascertainment of outcomes).

Fourth, the results of each study were categorized to indicate whether a treatment led to: 1) improvement; 2) no improvement; or 3) no difference in participants’ outcomes. However, the decisions used to make the judgements are not clear and cannot be critically appraised nor replicated. Specifically, what consideration was given to statistical significance versus clinical importance and to within- versus between-group differences? Also, the main descriptive results presented in data extraction tables (Tables 6–10) only provide a general summary of the results and the necessary information to interpret the results are lacking (e.g., control interventions, effect size, statistical significance, precision of estimates, clinical importance, etc.). We could not identify any supplemental tables providing such information about control interventions and effect size.

## Conclusions

Based on our concerns, we caution readers about the poor internal validity of the review and suggest that its recommendations should not be used to inform the clinical management of pediatric patients.

## Data Availability

Not applicable.

## Abbreviations

SIGN: The Scottish Intercollegiate Guidelines Network
PRESS: The Peer Review of Electronic Search Strategies
